# Relationship between fatty liver change and nutritional status after total gastrectomy in gastric cancer patients: a retrospective study

**DOI:** 10.1186/s12893-021-01324-x

**Published:** 2021-08-14

**Authors:** Naohiko Nakamura, Shinichi Kinami, Jun Fujita, Daisuke Kaida, Yasuto Tomita, Takashi Miyata, Tomoharu Miyashita, Hideto Fujita, Nobuhiko Ueda, Hiroyuki Takamura

**Affiliations:** Department of Surgical Oncology, Kanazawa Medical University Hospital, 1-1 Daigaku, Uchinada, Kahoku, Ishikawa 920-0293 Japan

**Keywords:** Gastric cancer, Fatty liver, Total gastrectomy

## Abstract

**Background:**

The relationship between chronological nutritional changes and development of fatty liver after total gastrectomy (TG) in gastric cancer (GC) patients is still unclear. This study aimed to evaluate relationship between development of fatty liver and chronological changes of nutritional parameters during 12 months after TG.

**Methods:**

We retrospectively analyzed medical records of 59 patients with GC who underwent TG at the Kanazawa Medical University Hospital between January 2009 and December 2017. We defined fatty liver change as a mean liver-to-spleen attenuation ratio (L/S ratio) of less than 1.2 in the computed tomography images at 12 months after TG and divided the patients into fatty liver (FL) and non-FL groups from the L/S ratio. We analyzed serum levels of total protein and albumin, and psoas muscle index (PMI) before TG and at 6 and 12 months after TG in the non-FL and FL groups.

**Results:**

Six patients showed an L/S ratio of less than 1.2 at 12 months after TG and were included into FL group. There was no significant difference between the groups in serum parameters, L/S ratio, and PMI before TG. In the FL group, the mean levels of total protein and albumin decreased after TG and were significant lower at 6 months, compared with the non-FL group. And then, these levels in the FL group recovered at 12 months. In contrast, the mean levels of total protein and albumin in the non-FL group did not decrease below the preoperative levels throughout the year after surgery. As with laboratory parameters, all patients in the FL group showed decrease of PMI at 6 months after TG. This proportion was significantly higher than that in the non-FL group (100% vs. 40.8%, P = 0.006).

**Conclusions:**

We evaluated that the patients with fatty liver occurring after TG had significantly lower levels of serum nutritional parameters and skeletal muscle index at 6 months, not but 12 months, after TG.

## Background

Gastric cancer (GC) is the fifth most common malignancy and the third most common cause of cancer mortality worldwide [1]. Although early GC is curable, advanced GC is still associated with poor survival, and the curative treatment consists of gastrectomy combining with preoperative chemotherapy [2, 3]. Total gastrectomy (TG) is mandatory to achieve curative resection of tumors invading the upper part of the stomach, especially widespread advanced GC or scirrhous type tumor that show higher risk of recurrence and poor prognosis. Therefore, most of advanced GC patients with TG need to undergo adjuvant chemotherapy for preventing postoperative recurrences. On the other hand, TG results in manifestations associated with nutritional disturbances, including weight loss, hypoalbuminemia, and vitamin deficiencies [4, 5]. Nutritional disturbances after TG could sometimes lead deterioration of general condition and other organ function. GC patients who become intolerance in postoperative chemotherapy, especially during first 1 year after surgery, due to nutritional disturbances after TG may result in exhibiting poor prognosis. Since, it is very important to maintain the chronological nutritional condition after TG and understand the nutritional influences to other organs in GC patients.

Fatty liver has become widespread and is mainly associated with the increasing prevalence of obesity [6]. In contrast, postoperative fatty liver change in patients who have undergone pancreatoduodenectomy is well known [7]. Fatty liver change after pancreatoduodenectomy would differ from fatty liver associated with obesity and cause by poor nutritional status [8, 9]. Similar to fatty liver associated with pancreatoduodenectomy, it is opposed that poor nutritional status after TG would influence hepatic metabolism and lead development of fatty liver. Recently, risk factors for fatty liver after TG have been identified, and fatty liver after TG has become increasingly problematic in the recent situation that multiple anticancer agents are used to adjuvant chemotherapy for advanced GC [10]. However, the relationship between chronological nutritional changes and development of fatty liver after TG is still unclear and further studies are needed to evaluate the mechanism of postoperative fatty liver in GC patients with TG. This study aimed to investigate fatty liver change and nutritional status after TG in GC patients and to evaluate relationship between development of fatty liver at 12 months after TG and chronological changes of nutritional parameters during 12 months after TG.

## Materials and methods

### Patients

A retrospective analysis was performed using medical records of 59 patients with GC who underwent TG at the Kanazawa Medical University Hospital between January 2009 and December 2017. Pre-operative clinical data, such as patient gender, age, and body mass index (BMI), were collected from the records of our hospital. We also extracted the results of the blood examination before surgery, which included information on serum levels of total protein, albumin, aspartate aminotransferase (AST), alanine aminotransferase (ALT), and neutrophil/lymphocyte ratios (NLR) in differential white blood cells. Based on pathological examination from resected specimen, tumor differentiation was pathologically assessed and patients were staged using the 8th edition of the UICC [11], according to the extent of lymph node metastasis (N) and distant metastasis (M). Patients with liver cirrhosis associated with hepatitis virus or alcohol, and patients with metabolic liver diseases were excluded in this study. Informed consent was obtained from all patients verbally. We evaluated surgical outcome such as lymphadenectomy, curability, operation time, and bleeding during operation. Surgical complications were classified according to the Clavien–Dindo classification [12]. This study was approved by the Medicine Ethics Committee of Kanazawa Medical University. We obtained written informed consent from all patients.

### Definition of fatty liver after TG

Computed tomography (CT) images were obtained with a 64 multidetector CT scanner before the surgery and at 6 and 12 months after surgery. For each patient, the average CT attenuation values of a region in the right and left liver lobes, and two regions in the spleen were measured. Each region of interest was a circular area with a diameter of 20 mm. The mean liver-to-spleen attenuation ratio (L/S ratio) on CT was calculated. Fatty liver change after TG was defined as an L/S ratio of less than 1.2 in the CT images at 12 months after TG [13]. We divided the patients into fatty liver (FL) and non-FL groups from the L/S ratio at 12 months after TG.

### Treatments

The choice of surgical procedure was made on a case-by-case basis, taking into account the status of the primary tumor (size, number, and location), and the general condition of the patient. Generally, we performed TG with D2 lymphadenectomy for advanced GC patients. Surgical curability (R0) was pathologically confirmed by a negative finding for residual tumor at the resected margin. Postoperative adjuvant chemotherapy for advanced GC consisted of S-1 monotherapy or S-1 combined with another drug. Patients with residual tumor, metastases, or postoperative recurrences underwent systemic chemotherapy according to the Japanese GC treatment guidelines [14].

### Evaluations: nutritional parameters and psoas muscle index after TG

We analyzed serum levels of total protein and albumin, and NLR at 6 and 12 months after TG in the non-FL and FL groups and assessed chronological changes in these nutritional parameters after TG. In addition, psoas muscle index (PMI) was applied as CT-determined skeletal muscle index. Both psoas muscle areas were calculated from a single CT image at the level of third lumber vertebrate (L3). PMI determined total psoas muscle area normalized to the squared patient height (cm^2^/m^2^). We assessed PMI before TG and at 6 and 12 months after TG in the non-FL and FL groups.

### Statistical analysis

Data were expressed as n (%) or mean (± standard deviation). Continuous variables and categorical variables were compared using the Student’s t-test and the χ^2^ test, respectively. All *P*-values were two-sided, and differences with a *P* < 0.05 were considered as statistically significant. The JMP software version 8.0 (SAS Institute, Cary, NC, USA) was used for all statistical analyses.

## Results

### Patient characteristics

In 59 patients, six patients showed an L/S ratio of less than 1.2 at 12 months after TG and were included into FL group. 53 others were included into non-FL group. Clinicopathological characteristic in the both groups was shown in Table 1. The mean age in the non-FL and FL groups was 68.4 and 63.0 years old, respectively. There was no significant difference between the groups in BMI, serum parameters, L/S ratio, and PMI before TG. NLR in the FL group was significantly higher than that in the non-FL group. The proportion of patients with postoperative chemotherapy was 62.3% in the non-FL group and 83.3% in the FL group (*P* = 0.31). Regarding with surgical outcomes and pathological findings (Table 2), significant difference was not observed between the groups.

**Table 1:** Patient characteristics

	Non-FL group (n = 53)	FL group (n = 6)	*P* value
Gender (male)	41 (77.3%)	4 (66.7%)	0.56
Age	68.4 (± 1.3)	63.0 (± 3.8)	0.18
BMI	23.0 (± 0.5)	23.9 (± 1.5)	0.58
Total protein (g/dl)	6.7 (± 0.1)	6.5 (± 0.3)	0.47
Albumin (g/dl)	3.9 (± 0.1)	3.7 (± 0.2)	0.28
AST (IU/l)	20.4 (± 0.6)	20.2 (± 1.9)	0.91
ALT (IU/l)	18.0 (± 1.0)	19.3 (± 3.0)	0.68
NLR	2.4 (± 0.2)	4.2 (± 0.6)	0.005
L/S ratio	1.33 (± 0.03)	1.23 (± 0.08)	0.12
PMI^a^	17.2 (± 0.7)	18.7 (± 2.2)	0.53
Postoperative chemotherapy (+)	33 (62.3%)	5 (83.3%)	0.31

Values are in n (%) or mean (± standard deviation)

*BMI* Body mass index, *AST* aspartate aminotransferase, *ALT* alanine aminotransferase, *NLR* neutrophil/lymphocyte ratios, *L/S ratio* liver-to-spleen attenuation ratio, *PMI* psoas muscle index

^a^PMI was calculated by CT image as total psoas muscle area at the level of third lumber vertebrate normalized to the squared patient height (cm^2^/m^2^)

**Table 2:** Surgical outcomes

	Non-FL group (n = 53)	FL group (n = 6)	*P* value
Pathological stage^a^			
pT > 3	28 (52.8%)	3 (60.0%)	0.79
pN > 1	25 (47.2%)	3 (60.0%)	0.61
pStage I/II	31 (58.5%)	3 (60.0%)	0.95
Tumor differentiation (poorly or signet)	28 (52.8%)	2 (33.3%)	0.37
Lymphadenectomy (D2)	17 (32.1%)	2 (33.3%)	0.95
Curabirity (R0)	43 (81.1%)	5 (83.3%)	0.90
Operation time (min)	292 (± 11)	298 (± 32)	0.86
Bleeding (ml)	280 (± 51)	270 (± 151)	0.95
Postoperative complication (+)^b^	11 (20.8%)	0 (0%)	0.27

Values are in n (%) or mean (± standard deviation)

^a^The 8th edition of the International Union Against Cancer (UICC) tumor-node-metastasis (TNM) classification [11]

^b^Postoperative complication was defined as more than grade III according to the Clavien–Dindo classification [12]

### Postoperative chronological change of L/S ratio in the non-FL and FL groups

**Fig. 1:**
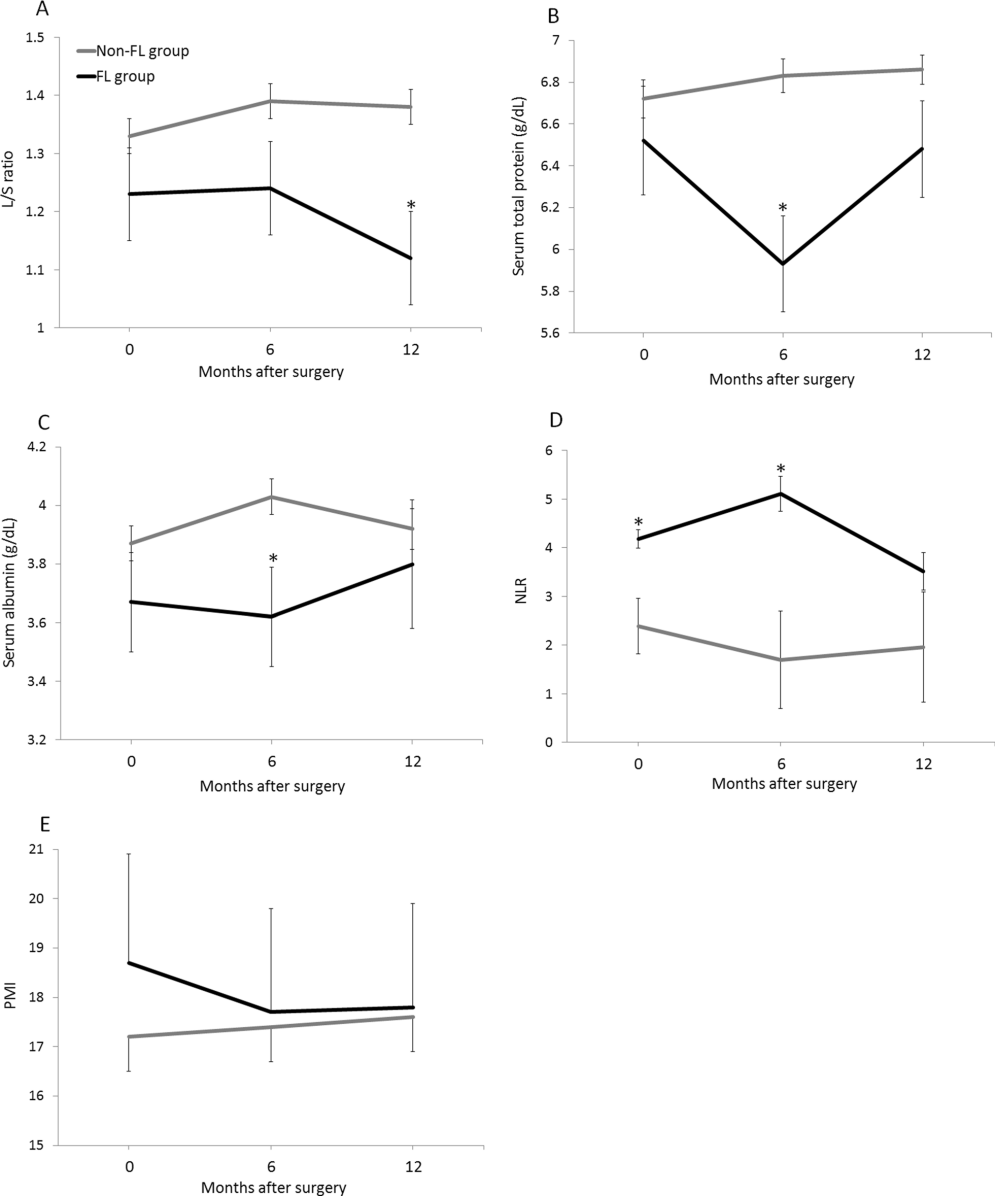
Postoperative chronological change of the clinical parameters in the non-FL and FL groups. **A** Mean liver-to-spleen attenuation ratio (L/S ratio). **B** Mean level of serum total protein. **C** Mean level of serum albumin. **D** Mean neutrophil/lymphocyte ratio (NLR). **E** Mean psoas muscle index (PMI). *P value < 0.05, compared to the non-FL group

There was no significant difference in the L/S ration before surgery between groups. Interestingly, the L/S ratio in the FL groups did not decrease at 6 months, but significantly declined from 6 to 12 months (Fig. 1A). The L/S ratio of 12 months in the non-FL and FL groups were 1.38 and 1.12, respectively.

### Postoperative chronological change of nutritional parameters in the non-FL and FL groups

In the FL group, the mean level of serum total protein decreased after TG and was significant lower at 6 months, compared with the non-FL group. This level in the FL group recovered at 12 months and there was no significant difference between the groups. In contrast, the mean level of serum total protein in the non-FL group did not decrease below the preoperative level throughout the year after surgery (Fig. 1B). As with the results in total protein, the mean level of serum albumin in the FL group decreased until 6 months and then increased at 12 months after TG (Fig. 1C).

### Postoperative chronological change of NLR in the non-FL and FL groups

Although patients in the FL group higher NLR before surgery, NLR in the FL group further increased to 5.11 and was significantly higher at 6 months than that in the non-FL groups. After 6 months NLR in the FL group decreased until the preoperative level and significant difference was not observed at 12 months between the groups (Fig. 1D).

### Postoperative chronological change of PMI in the non-FL and FL groups

The mean PMI in the FL group decreased from before TG to 6 months after TG, but that in the non-FL group did not decrease during the year after TG (Fig. 1E). Although significant difference did not overserved at 6 months between the groups, all patients in the FL group showed decrease of PMI at 6 months from before TG. This proportion was significantly higher than that in the non-FL group (100% vs. 40.8%, *P* = 0.006). In contrast, The proportion of patients who showed decrease of PMI from 6 to 12 months was 44.0% in the non-FL group and 33.3% in the FL group (*P* = 0.62).

## Discussion

In this study, we assessed relationship between postoperative fatty liver change and chronological nutritional parameters after TG in GC patients. To our knowledge, no clinical studies have investigated a chronological influence of postoperative nutritional condition in development of fatty liver after TG. We analyzed chronological change of serum nutritional parameters and skeletal muscle index during 1 year after TG. In the patients with fatty liver occurring after 12 months of TG, the serum levels of total protein and albumin chronologically decreased until 6 months and became significantly lower at 6 months after TG, compared with those in the patients without fatty liver. However, these serum levels in the patients with fatty liver recovered to preoperative state at 12 months after TG. The previous report regarding with fatty liver after TG showed that there was no significant difference in serum levels of total protein and albumin at postoperative 12 months between the patients with fatty liver and without fatty liver [10]. This is compatible to our results at 12 months, but these parameters at 6 months after TG have not previously evaluated. In addition, the PMI in the patients with fatty liver decreased towards 6 months after TG and all the patients with fatty liver showed decreased PMI at 6 months after TG. Combining with these results, deterioration of nutritional condition at 6 months but not 12 months could associate with development of fatty liver at 12 months after TG. Moreover, there may be a time lag between the deterioration of nutritional status and occurrence of fatty liver because the L/S ration at 6 months in the FL group did not decline despite of deterioration of nutritional condition at 6 months.

Adjuvant chemotherapy was previously reported as a risk factor for fatty liver development after TG [10]. Although it is assumed that the surgical outcomes including postoperative complications or performing postoperative chemotherapy may affect postoperative nutritional condition, no significant difference was found in these factors between the groups in this study. On the other hand, preoperative NLR in the FL group was significantly higher than that in the non-FL group. Furthermore, the NLR in the FL group elevated at 6 months after TG. The NLR is a simple index that could be calculated in a routine examination as the non-invasive biomarker of surgical stress [15]. The elevated NLR have been previously reported to be related to the poor prognosis of GC, colorectal, and lung cancer [16, 17]. Although the reason why the preoperative NLR in the FL group was higher is unclear, prolonged postoperative stress may affect exhaustion of nutrition condition at 6 months after TG.

The PMI is one of the skeletal muscle indexes that can be easily and rapidly assessed by CT imaging, and significant correlation was previously found between sarcopenia and PMI [18]. In the patients with fatty liver after TG, the PMI as well as nutritional serum parameters decreased at 6 months. Nutritional support is very important for countering sarcopenia [19]. Since, persistence of malnutrition after TG can be considered as one of the causes of decreased PMI at 6 months. Our results suggest that loss of skeletal muscle mass coincided with malnutrition would associated with the development of fatty liver after TG. GC patients who undergo TG for advanced GC often have opportunity to undergo adjuvant chemotherapy or systemic chemotherapy for postoperative recurrence. Liver fibrosis associated with fatty liver was previously reported to be a risk factor of hematological toxicity of chemotherapy in colorectal cancer patients [20]. Therefore, it is essential for prevention of fatty liver occurring at 12 months to perform immediate postoperative nutritional intervention and rehabilitation in GC patients with TG, especially advanced GC patients, in order to keep nutritional condition and muscle mass until 6 months after TG. Prevention of fatty liver occurring after surgery could lead achievement of multidisciplinary therapy and improvement of postoperative prognosis in GC patients.

In our analysis, the incidence of fatty liver after TG for GC was 10.2%. This incidence rate was similar or lower than that associated with pancreatoduodenectomy, which is reported to be 8–40% [21]. On the other hand, a recent study reported that 4.85% GC patients who underwent TG exhibited fatty liver [10]. The authors diagnosed fatty liver by using CT attenuation values and the incidence of fatty liver was lower than that in our analysis. We using an L/S ratio at CT images and defined fatty liver as L/S ratio of less than 1.2. The difference in the method and definition of fatty liver could affect the incidence of fatty liver after TG. Generally, fatty liver is strongly associated with obesity and other metabolic disease, including type 2 diabetes mellitus and dyslipidemia, and is also one of the most common causes of chronic liver disorders and can lead to liver cirrhosis or hepatocellular carcinoma [22, 23]. In contrast, it has been reported that fatty liver after pancreatoduodenectomy is mainly associated with malnutrition and pancreatic exocrine deficiency [24]. Although advanced GC patients often exhibit malnutrition before surgery, there is no significant difference in preoperative nutritional parameters between the FL and non-FL groups in the present study. These results was compatible to the results in the previous report that GC patients with fatty liver after TG did not have lower serum levels of total protein and albumin before surgery [10]. Present study indicated that malnutrition that was caused by TG at 6 months after surgery could be involved in the development of fatty liver.

While we believe that our study brings a new perspective to the relationship between chronological nutritional condition and fatty liver change after TG in GC patients, it also has certain limitations. This was a retrospective study performed at a single institution and the sample size was small. Thus, to confirm the mechanism in development of fatty liver after TG, further work with a prospective cohort study in multiple institutions is warranted.

## Conclusions

We evaluated that the patients with fatty liver occurring after TG had significantly lower levels of serum nutritional parameters and skeletal muscle index at 6 months, not but 12 months, after TG. Our findings suggest that malnutrition and muscle mass loss of postoperative 6 months that was caused by TG could be involved in the development of fatty liver.

## Data Availability

All data are available without restriction. Researchers can obtain data by contacting the corresponding author.

## Abbreviations

GC: Gastric cancer
TG: Total gastrectomy
BMI: Body mass index
AST: Aspartate aminotransferase
ALT: Alanine aminotransferase
NLR: Neutrophil/lymphocyte ratios
UICC: International Union Against Cancer
TNM: Tumor-node-metastasis
CT: Computed tomography
L/S ratio: Liver-to-spleen attenuation ratio
FL: Fatty liver
PMI: Psoas muscle index
